# Focal, Extranodal Primary Polymorphous Hemangioendothelioma Treated With Endovascular Embolization and Resection Surgery

**DOI:** 10.7759/cureus.19756

**Published:** 2021-11-19

**Authors:** Esteban Ramírez-Ferrer, Francisco Perez, Alberto Caballero, William Mauricio Riveros, Luis Alejandro Osorio-Bohorquez

**Affiliations:** 1 Neurological Surgery, Center for Research and Training in Neurosurgery (CIEN), Bogotá, COL; 2 Neurological Surgery, Hospital Universitario de La Samaritana, Bogotá, COL; 3 Neurological Surgery, Rosario University School of Medicine, Bogotá, COL; 4 Neurological Surgery, Hospital Universitario Mayor de Méderi, Bogotá, COL; 5 Neurological Surgery, Center for Research and Training In Neurosurgery (CIEN), Bogotá, COL; 6 Neurological Endovascular Surgery, Hospital Universitario de La Samaritana, Bogotá, COL; 7 Neurological Endovascular Surgery, Center for Research and Training in Neurosurgery (CIEN), Bogotá, COL

**Keywords:** tumor-free resection margins, polymorphous hemangioendothelioma, extranodal, arteriography, endovascular embolization, rare vascular tumor, resection surgery

## Abstract

A male 28-year-old patient complained of a dorsal mass that has been increasing in size in the last six months. The mass was painful, soft, no mobile, and no neurological symptoms or signs were documented. A vascular-type tumor was suspected and endovascular followed by open surgical resection was indicated.

Histopathological revealed a rare case of an adult with a primary extranodal polymorphous hemangioendothelioma. Total resection was confirmed by tumor-free resection margin. The postoperative course was uneventful.

Polymorph hemangioendothelioma is a rare vascular tumor. Preoperative endovascular embolization is recommended given the high vascular features of the lesion and, therefore, the high rate of bleeding during surgery, to achieve complete resection.

## Introduction

Hemangioendothelioma is a rare vascular tumor that was first described in 1982 [1]. It is a tumor with a biological borderline behavior as an intermediary pathology between benign hemangioma and malignant angiosarcoma [2]. Also, it is divided into different subtypes relying on the cellular features. These subtypes are: epithelioid, papillary intralymphatic, retiform, kaposiform, pseudomyogenic, composite, and polymorphous; the last being the rarest variant.

The first three cases of polymorphous hemangioendothelioma (PH) were described in 1992 in an analysis of 39 cases of primary vascular lymph node tumors [3]. Until 1999, six cases had been described [4]. A review of the literature revealed that, up to date, less than 10 cases of properly called PH can be found in the literature [5-9], most of them from the lymph nodes and a rare case from maxillary soft tissue PH [8]. Only a few cases of primary extranodal presentation have been described in the literature [4].

Given the small number of cases found, the natural history of the disease has not been elucidated. However, the potential for PH to metastasize and recur has been described [2,6,8] and, therefore, total resection has been proposed as the first line of treatment [1]. Radiation [2] and immunotherapy [3] have been proposed without sufficient evidence. Also, the utility of endovascular embolization followed by open resection has not been described in the context of a vascular malignancy with a high bleeding rate during open surgery to achieve total resection.

(This article was previously posted to the Research Square preprint server on February 1, 2021)

## Case presentation

A 28-year-old male patient presented to the outpatient clinic due to a “lump” in his dorsal back that had been increasing in size in the previous six months. He did not report any weight loss, fever, or neurologic deficit. The patient had been referred to a different hospital where an open biopsy procedure was performed two months earlier but due to significant blood loss, the procedure had to be suspended. The histopathology result described a lesion compatible with hemangioma. Since then, the patient had noted a significant size increase. The patient had no other medical conditions and no medication use. Physical examination showed a dorsal 10 cm mass, non-mobile, slightly painful, with no erythema or secretions associated. The rest of the examination was unremarkable.

**Figure 1:**
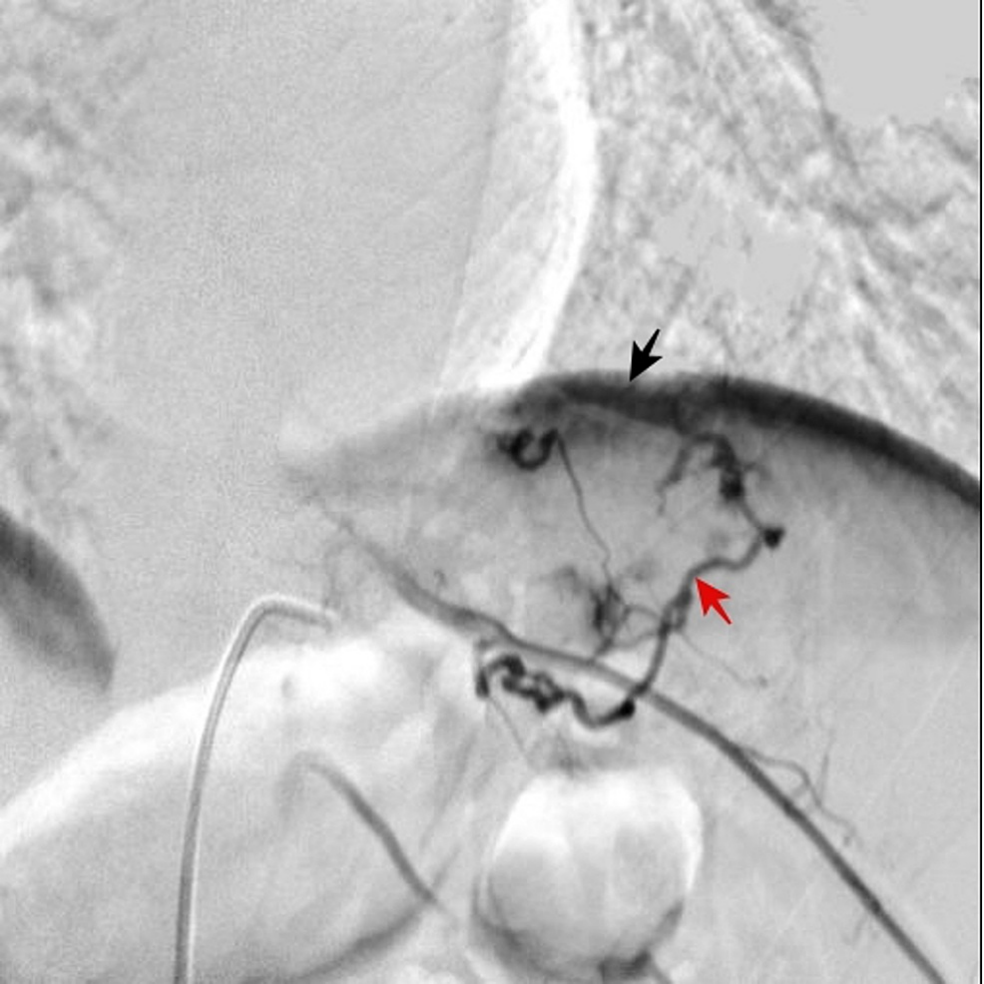
Pre-embolization spinal arteriography Selective catheterization of radiculomedullary branches was performed through the T10 and T11 segmental artery. Mass arterial supply was documented through T10 (black arrow) and T11 (red arrow) radiculomedullary arteries.

**Figure 2:**
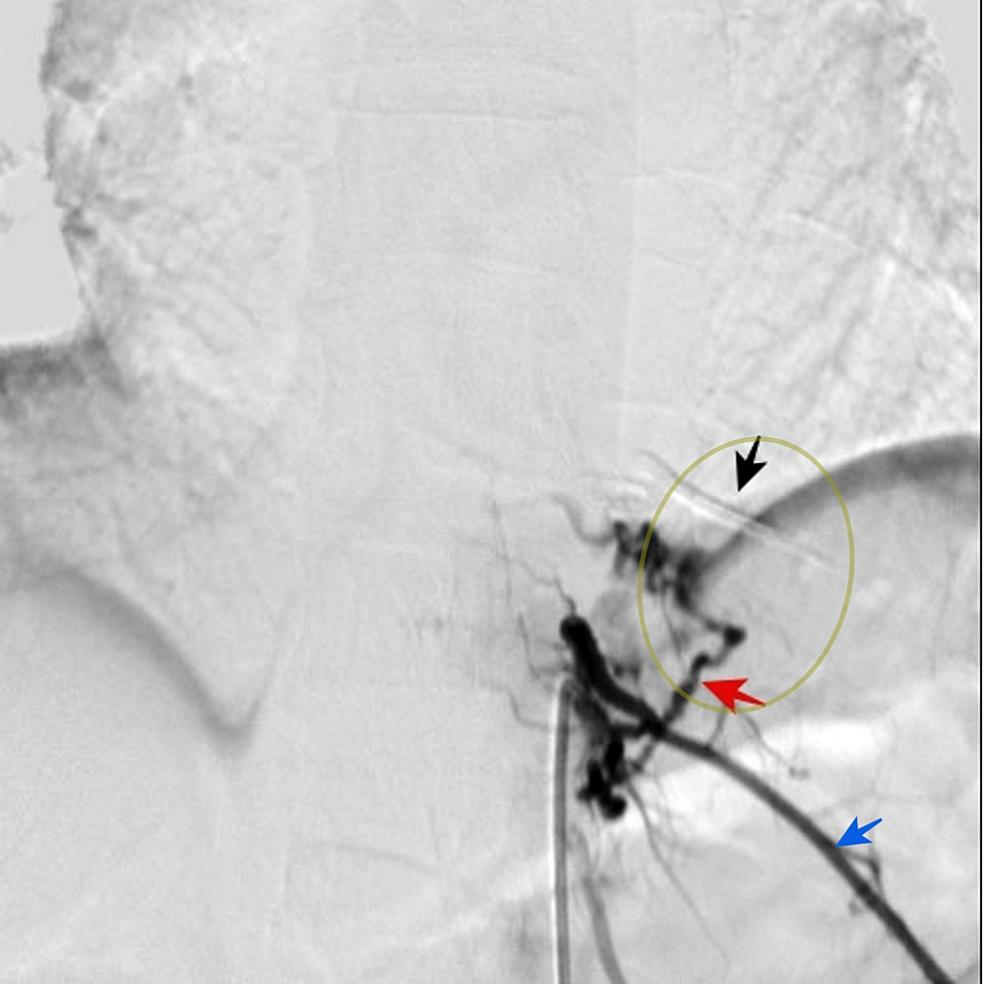
T10 and T11 intersegmental arteries arteriography Selective embolization of the right T10 radiculomedullary artery is performed (black arrow) through intersegmental artery catheterization. T11 radiculomedullary artery embolization was not performed because multiple branches were visualized (red arrow). Lateral branch of intersegmental T11 artery is showed (blue arrow). Aproximate location of tumor is indicated (olive circle)

A thoracic spine contrast-enhancing magnetic resonance imaging (MRI), revealed a dorsal mass at T8 level with contrast enhancement, compromising only soft tissues. The mass did not involve the vertebrae or medullary canal. No lymph node enhancement was documented. Given the blood loss history during the previous surgical procedure, spinal arteriography was indicated to assess mass arterial supply. The spinal arteriography (Figure 1) was performed with a Cobra catheter through the right femoral artery, ascending from T12 up to T5 radicular arteries. Artery blood supply to the dorsal mass was registered mainly from T10 segmentary arteries and, in less degree, by segmentary arteries of T11. Embolization of the T10 and T11 radiculomedullary branches was indicated. Selective catheterization of the T10-radiculomedullary branch was achieved, Adamkiewicz artery was not present at this level (confirmed by contrast injection), and coil-embolization was performed with awake patient. In contrast, T11-radiculomedullary embolization could not be done because multiple branches directing to the spinal cord were documented (Figure 2). Intraoperative neurophysiologic monitory was continuously performed without abnormalities. A 60% reduction of arterial supply was achieved.

**Figure 3:**
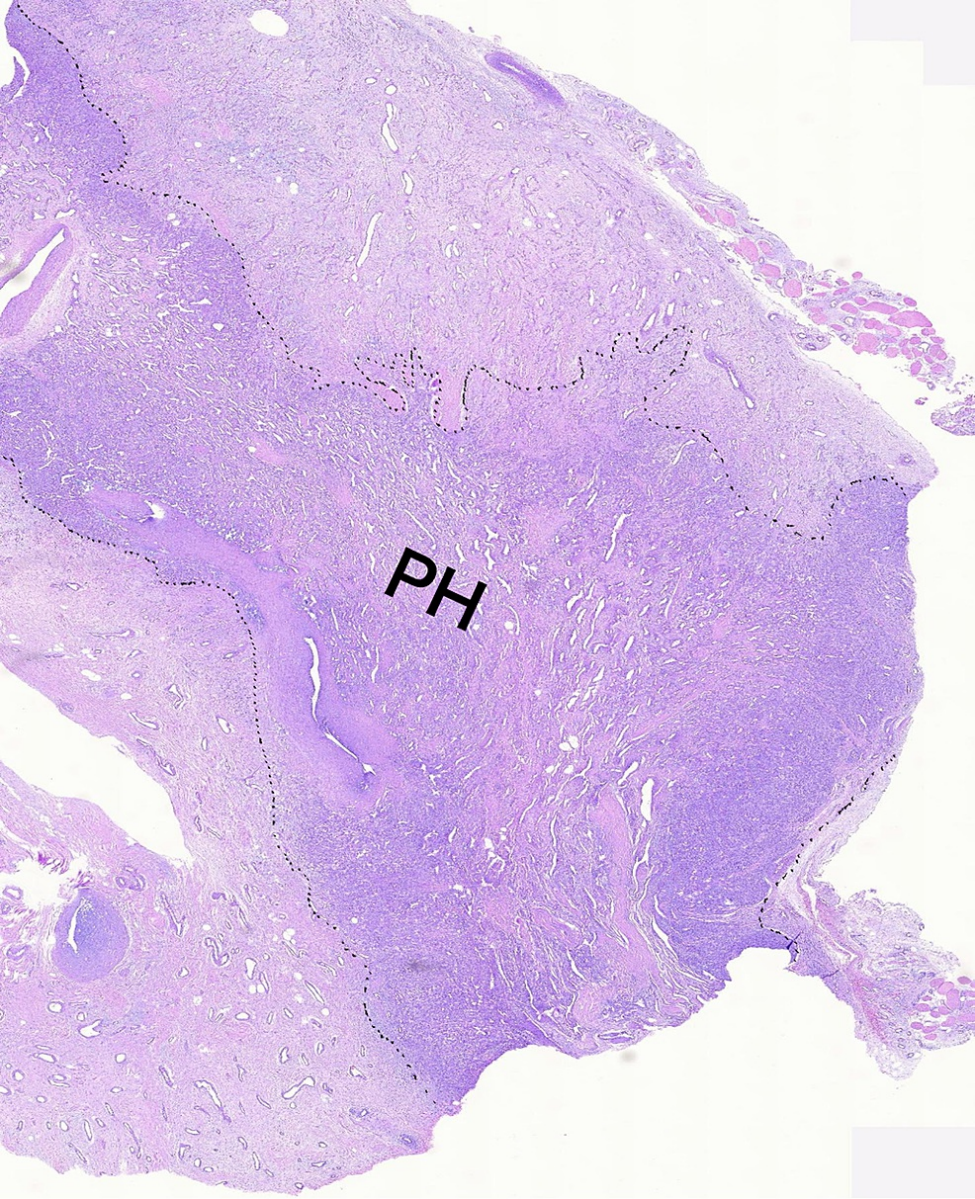
Histology of the tumor-free borders (dotted lines) mass PH: polymorphous hemangioendothelioma

**Figure 4:**
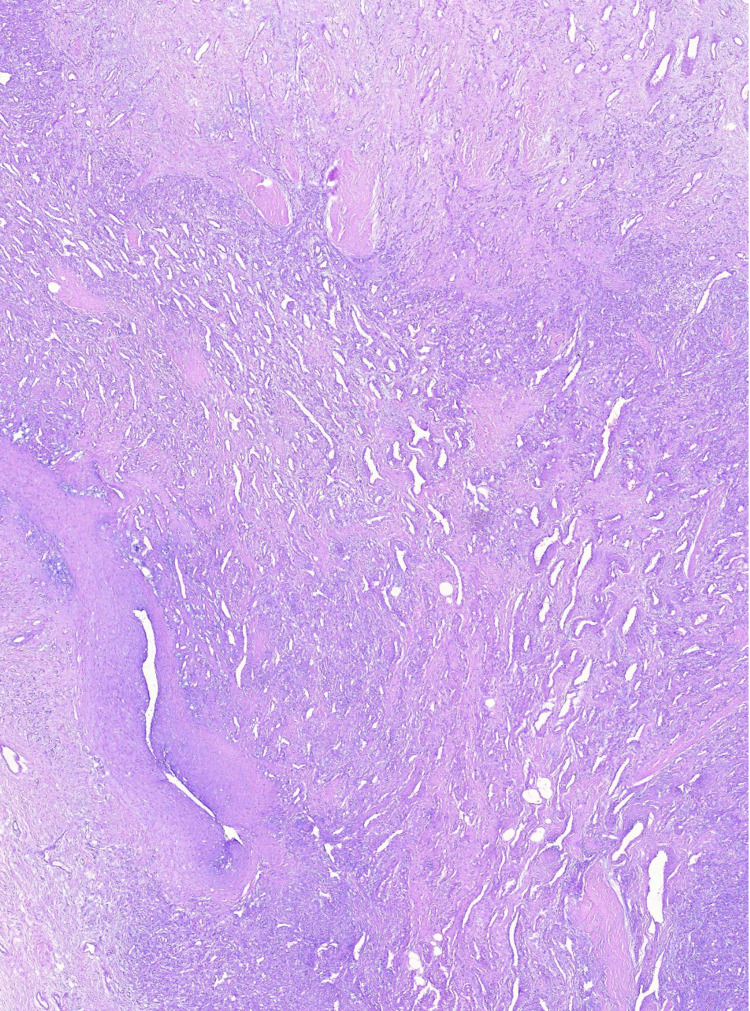
Tumor area with angiomatous and retiform growth pattern with areas composed of ovoid, polygonal, and spinal cells, with low mitotic activity.

One day after embolization, the patient was taken to open surgical resection. Dissection through the tumor plane was performed, the vascular supply from T10 and T11 radiculomedullary branches was sparse due to previous endovascular embolization. No significant bleeding occurred and complete resection of the lesion was achieved (Figure 3), which was confirmed with the histopathological report of tumor-free borders (Figure 3 and 4). The patient recovered without complications and was discharged 24 hours later.

## Discussion

Hemangiothelioma is a borderline vascular tumor between benign hemangioma and malignant sarcoma. Seven different subtypes of properly hemangioendothelioma have been described (Table 1).

**Table 1:** Hemangioendothelioma subtypes

Hemangioendothelioma subtype	Histopathology
Epithelioid	Round, oval, and polygonal cells with abundant pale eosinophilic cytoplasm embedded in a fibromyxoid or sclerotic stroma.
Papillary intralymphatic (Dabska tumor)	Central hyaline core lined by hobnail-like endothelial cells protruding into the lumina.
Retiform	Elongated arborizing vessels arranged in anastomosing pattern resembling rete testis.
Kaposiform	Several solid poorly circumscribed nodules, each constituted by a mixture of small capillaries and solid lobules of endothelial cells arranged in glomeruloid pattern.
Pseudomyogenic (sarcoma-like)	Poorly circumscribed and fascicular lesion with infiltrative borders composed of round or oval neoplastic cells.
Composite	varying combinations of benign, low-grade malignant, and high-grade malignant vascular components
Polymorphous	Spindled cell areas, polygonal epithelioid areas and variable vascular density

The PH natural history, rate of recurrence and potential to metastasize is not well known given the limited available reported cases in the literature. Likewise, the first-line treatment has not been standardized, but total resection is recommended. Additionally, immunotherapy and radiotherapy has been proposed as adjuvant treatment [1,2].

Given the vascular etiology of the PH, operative bleeding can be of concern and should be addressed. This case was approached with preoperative arteriography that revealed arterial supply from radicular T10 left artery and, in the absence of Adamkiewicz artery, embolization was indicated and could be performed without complication. Afterwards, open surgical resection was performed with low operative bleeding and complete resection with tumor-free edges was obtained. This is the first case of extranodal, primary PH treated with endovascular coiling followed by open resection, and showed exceptional results.

## Conclusions

PH is a rare vascular tumor. The classification and natural history depend on localization (nodal vs extranodal). The focal, extranodal primary polymorph hemangioendothelioma could be treated with a combined neurosurgical approach consisting of endovascular coil-embolization followed by resection surgery, which can enhance total resection, decreasing the risk of surgical complications as incomplete resection or bleeding.
